# The influence of different growth hormone addition protocols to poor ovarian responders on clinical outcomes in controlled ovary stimulation cycles: A systematic review and meta-analysis

**DOI:** 10.1097/MD.0000000000006443

**Published:** 2017-03-24

**Authors:** Xue-Li Li, Li Wang, Fang Lv, Xia-Man Huang, Li-Ping Wang, Yu Pan, Xiao-Mei Zhang

**Affiliations:** a Reproductive Medicine Center, Department of Obstetrics and Gynecology, Clinical Medical College, Yangzhou University, Northern Jiangsu Province Hospital, Yangzhou, China; b Department of Anesthesiology & Perioperative Medicine, The University of Texas MD Anderson Cancer Center, Holcombe Boulevard, TX; c Department of Biobank, Clinical Medical School of Yangzhou University, Northern Jiangsu Province Hospital, Yangzhou, China.

**Keywords:** clinical outcomes, growth hormone, in vitro fertilization, poor ovarian responders

## Abstract

**Background:**

Growth hormone (GH) is used as an adjuvant therapy in in vitro fertilization and embryo transfer (IVF-ET) for poor ovarian responders, but findings for its effects on outcomes of IVF have been conflicting. The aim of the study was to compare IVF-ET outcomes among women with poor ovarian responders, and find which subgroup can benefit from the GH addition.

**Methods:**

We searched the databases, using the terms “growth hormone,” “GH,” “IVF,” “in vitro fertilization.” Randomized controlled trials (RCT) were included if they assessed pregnancy rate, live birth rate, collected oocytes, fertilization rate, and implantation rate. Extracted the data from the corresponding articles, Mantel–Haenszel random-effects model, or fixed-effects model was used. Eleven studies were included.

**Results:**

Clinical pregnancy rate (RR 1.65, 95% CI 1.23–2.22), live birth rate (RR1.73, 1.25–2.40), collected oocytes number (SMD 1.09, 95% CI 0.54–1.64), MII oocytes number (SMD 1.48, 0.84–2.13), and E_2_ on human chorionic gonadotropin (HCG) day (SMD 1.03, 0.18–1.89) were significantly increased in the GH group. The cancelled cycles rate (RR 0.65, 0.45–0.94) and the dose of gonadotropin (Gn) (SMD –0.83, –1.47, –0.19) were significantly lower in patients who received GH. Subgroup analysis indicated that the GH addition with Gn significantly increased the clinical pregnancy rate (RR 1.76, 1.25–2.48) and the live birth rate (RR 1.91, 1.29–2.83).

**Conclusion:**

The GH addition can significantly improve the clinical pregnancy rate and live birth rate. Furthermore, the GH addition time and collocation of medications may affect the pregnancy outcome.

## 1 Introduction

Many different studies reported that the incidence of poor ovarian responders (POR) is increasing and vary from 9% to 24%. The problem of POR has been increased following the increasement of later marriage and childbearing in assisted reproductive technology (ART).^[1–4]^ POR has been related to several factors, including advanced female age, high body mass index, and history of ovarian and pelvic surgeries.^[5]^ However, the definition of POR was debatable and not unified for many years. According to Bologna Criteria^[6]^ in 2011, POR should be diagnosed as the result of the presence of at least 2 of the 3 features: age≥ 40 years or any other risk factor for POR, POR history (3 of fewer oocytes with ovulation induction), and low ovarian reserve test. Although the low successes, there are many intervention protocols that have been suggested to improve the outcome of IVF in poor responders, such as adding growth hormone as an adjuvant treatment to the stimulation protocols.^[7]^ Many studies show that GH plays an important role in granulose cell, which can promote ovarian steroid genesis and follicular development.^[8,9]^ The first report of GH role in POR which published 25 years ago is puzzling.^[10]^ Four meta-analysis assessed the value of GH addition in IVF. A meta-analysis by Kolibianakis et al^[11]^ had reported an increment in the clinical pregnancy rate and the live birth rate with the administration of GH in POR, however, the number of cases studied was too small. Kyrou et al^[12]^ found an improvement on the probability of pregnancy with GH addition on day 2 versus day 3 of embryo transfer. A meta-analysis showed that GH supplement increased serum estradio (E_2_) level on HCG day, Metaphase II (MII) oocyte number, 2PN number, and obtained embryo number,^[13]^ however there was no significant difference on clinical pregnancy rate. A 2003 Cochrane review thought that the GH role in IVF needed further research.^[14]^ The aim of this meta-analysis is compare IVF outcomes among women with POR who used GH or not, and find which subgroup can benefit from GH.

## 2 Materials and methods

This meta-analysis does not involve patients and, thus, do not require institutional review board approval. Databases including PubMed, Medline, Embase, and Cochrane Library were searched for reports published. The search terms were “growth hormone,” “GH,” “IVF,” “in vitro fertilization.” We also divided the included articles into 2 subgroups, 1 group was GH addition with Gn, and the other group was GH addition in the middle luteal phase, and then compared which subgroup could benefit from GH. In addition, the relevant studies were also searched in the references of selected articles and reviews.

Inclusion criteria were as follows: (1) the study population was POR or sub-optimal responders undergoing IVF or intracytoplasmic sperm injection (ICSI), with any ovarian stimulation protocol; (2) the selected articles were RCT; and (3) the reported outcomes were pregnancy rates, live birth number, cancelled cycles, collected oocytes number, MII oocytes number, implantation rate, fertilization rate, E_2_ on HCG day and dose of gonadotropin.

The abstracts of all studies by keywords search were screened by 2 investigators (XL and FL). The eligible abstracts were evaluated independently by 2 reviewers (XL and XH). Any disagreement between 2 reviewers was resolved through discussion. If the abstract of a study was eligible, then 2 investigators (XL and LW) read and judged the whole article carefully.

Data for methods (type of articles, purpose of intervention, method of allocation, inclusion criteria), participant characteristics (number of participants and age), interventions (dose of GH, and other stimulation protocols), and outcomes (pregnancy rates, live birth number, cancelled cycles, collected oocytes, MII oocytes number, implantation rate, fertilization rate, E_2_ on HCG day and dose of gonadotropin) were extracted by 2 reviewers (XZ and KL). Any disagreement between 2 reviewers was also resolved through discussion. Articles were also assessed for potential sources of bias, including the solution of randomization, allocation concealment, and blinding.

We used Review Manager 5.2 to analyze the results. Data are presented as mean ± standard deviation or number (%). Outcomes were sum up by cumulating risk ratio (RR) and 95% confidence intervals (CIs). *χ*^*2*^ test and *I*^2^ were used to assess the heterogeneity between studies. If the *I*^2^>50% or *P*<0.10 indicates significant heterogeneity, the Mantel–Haenszel random-effects model was used, otherwise, fixed-effects model was used.

## 3 Results

**Figure 1:**
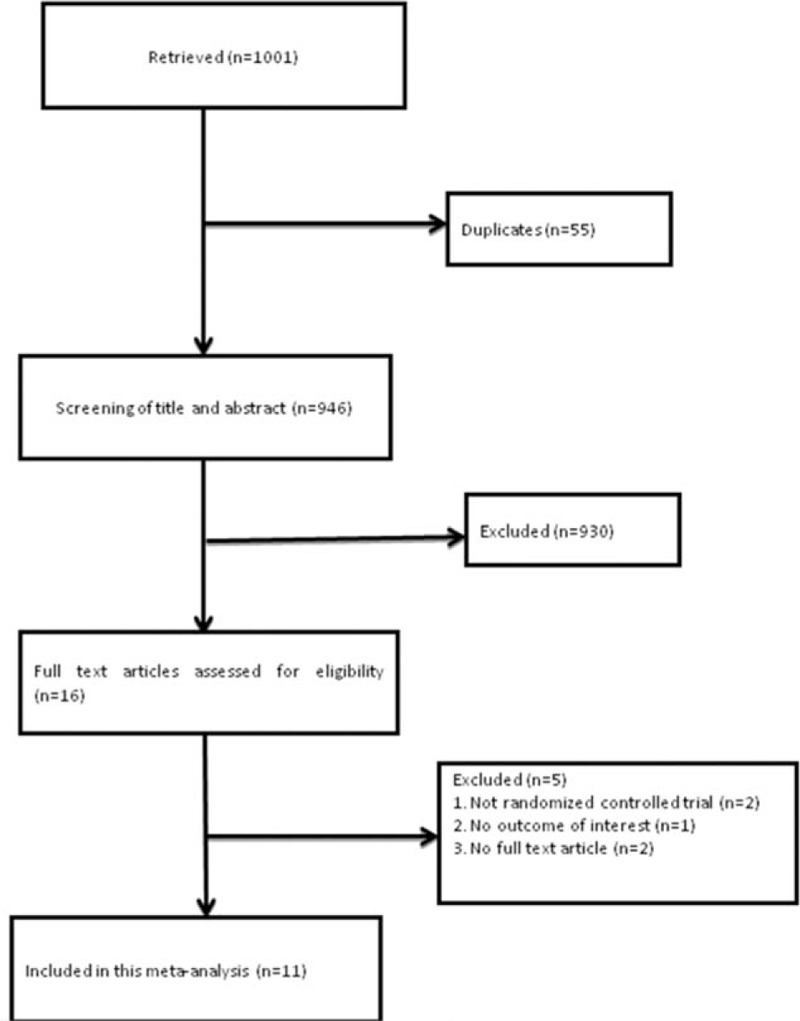
Flowchart of study selection.

**Figure 2:**
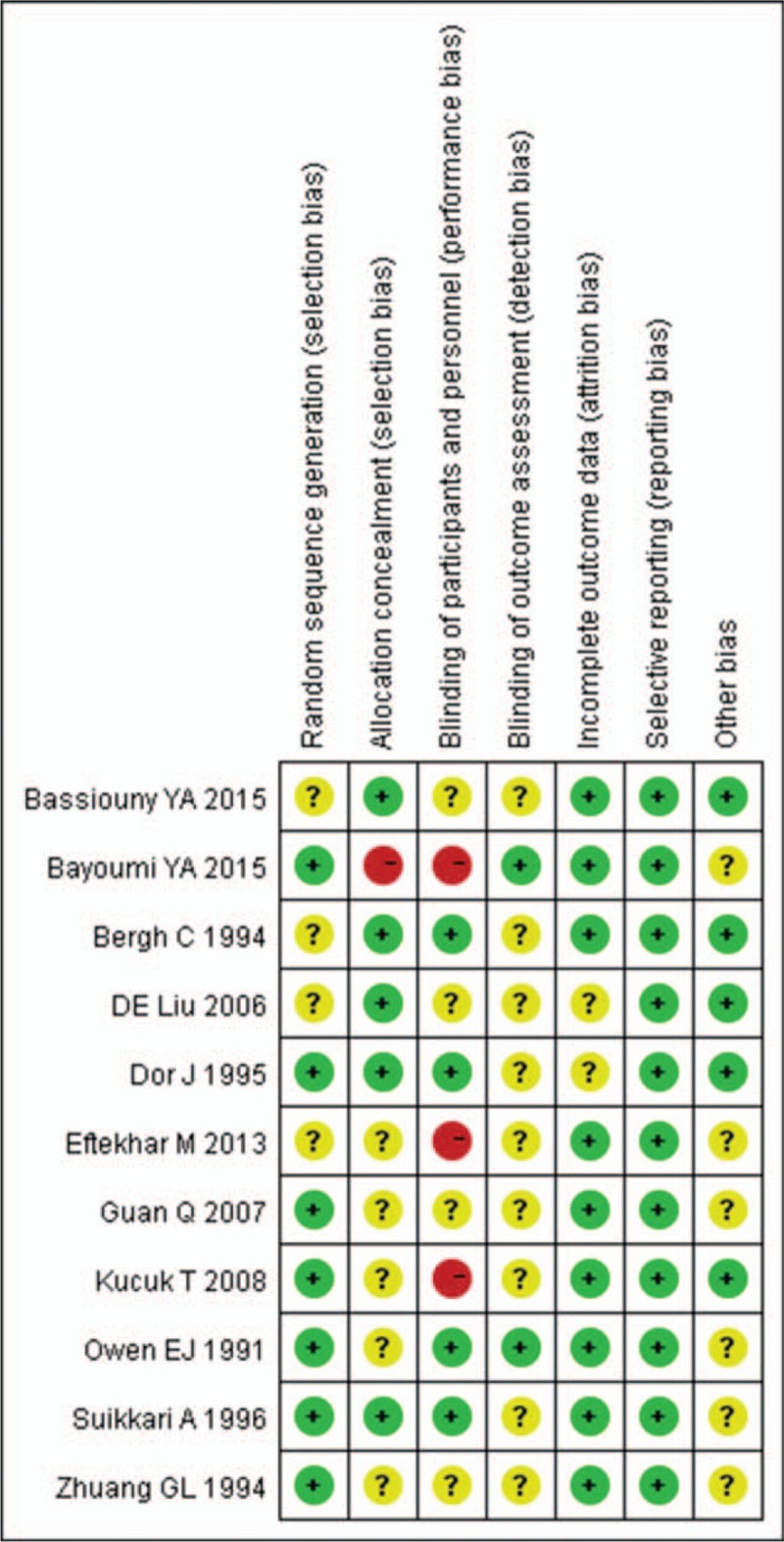
Quality assessments of included studies. ? = unclear, + = low risk, – = high risk.

A total of 16 articles were fully eligible, 2 articles were not RCT, 1 article was no outcome of interest, and 2 articles were no full text, so11 (663 patients) articles were included in this meta-analysis (Fig. 1, Table 1). The quality assessment of the included studies was presented in Fig. 2.

**Table 1:**
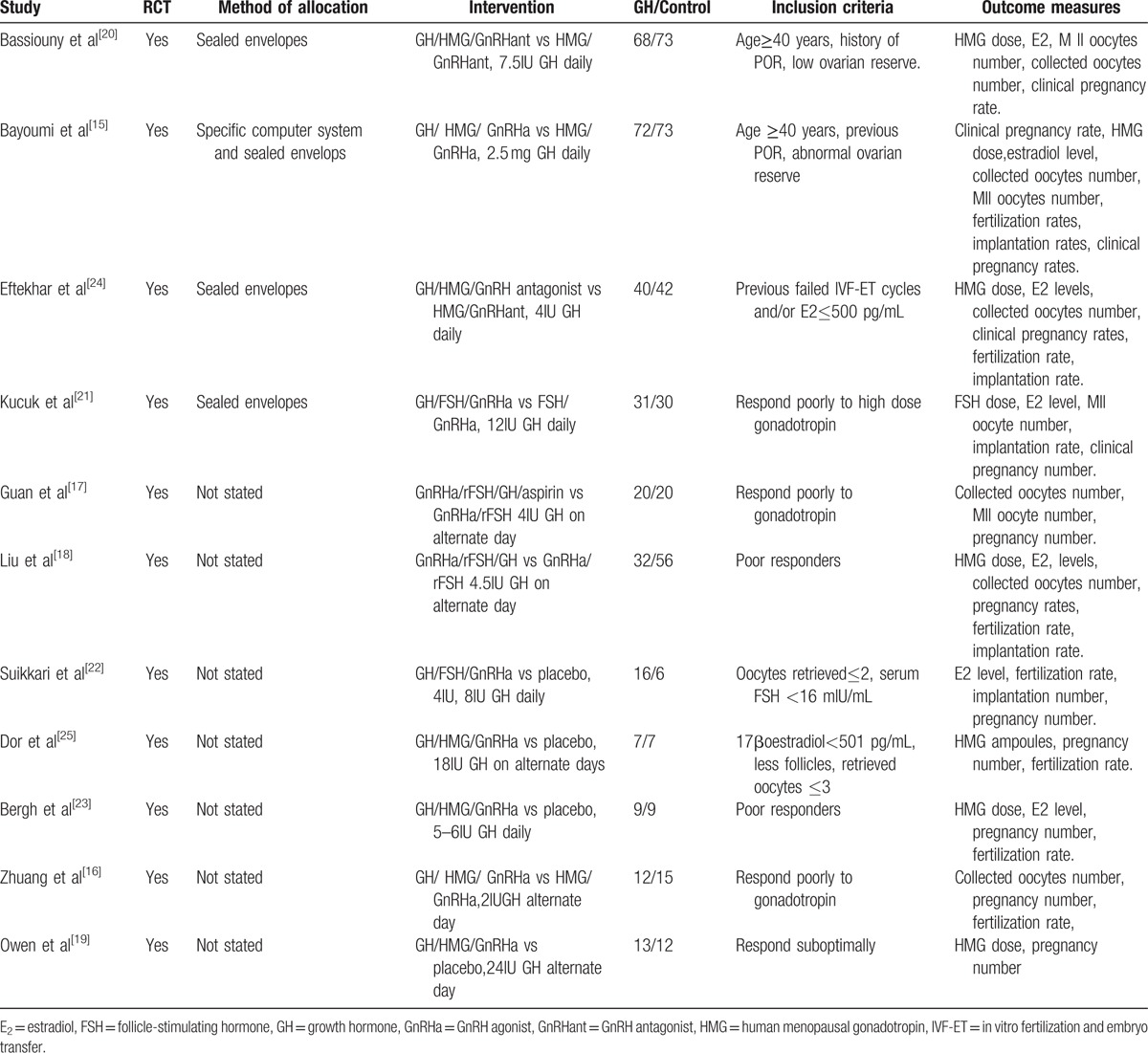
Included studies.

### 3.1 Pregnancy rate

**Figure 3:**
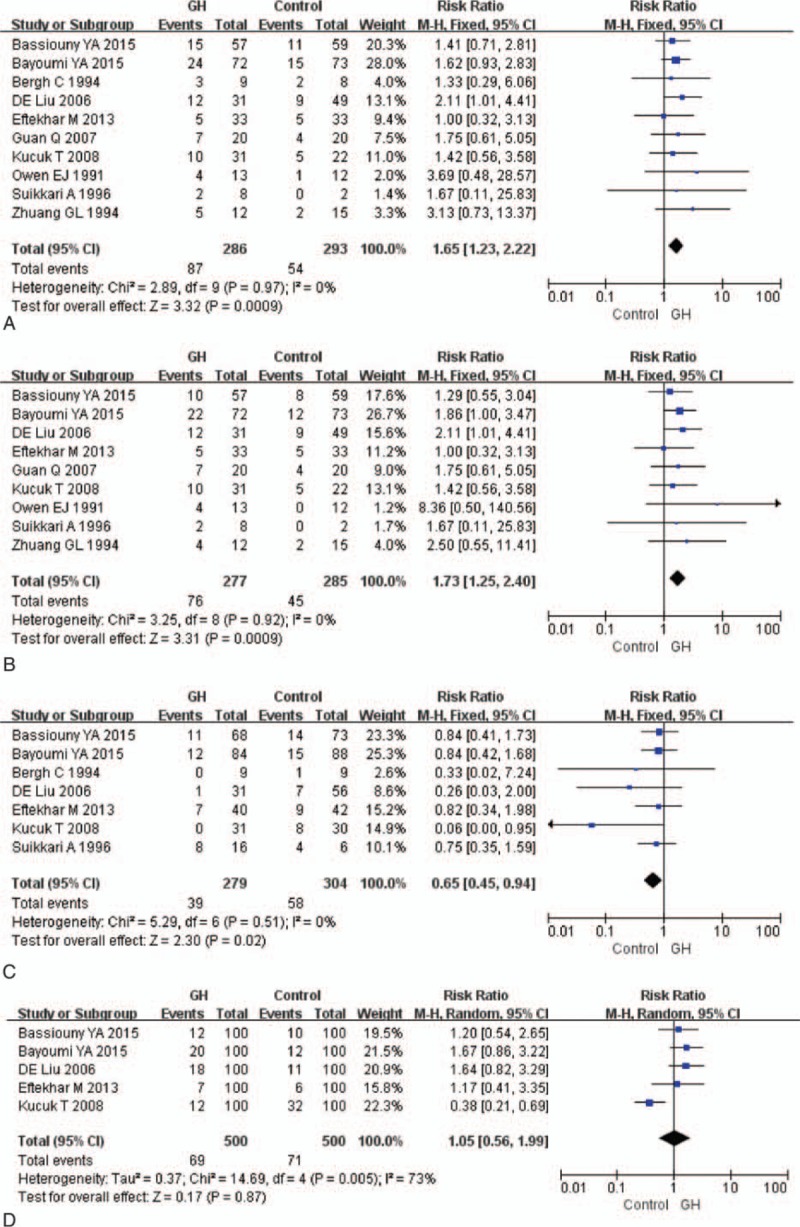
Forest plots for (A) clinical pregnancy rate, (B) live birth rate, (C) cancelled cycles rate, and (D) implantation rate. CI = confidence interval, GH = growth hormone.

All 11 studies, only 10 studies reported clinical pregnancy or clinical pregnancy rate, and were included in this meta-analysis (Fig. 3A). Six^[15,16,18,20–22]^ studies showed an increase of pregnancy rate among women who received GH, whereas the difference did not reach to statistical significance. A pooled result using fixed-effects model showed that clinical pregnancy rate (RR 1.65, 95% CI 1.23–2.22; *p *< 0.001) was significantly increased in the GH group. There was no heterogeneity between studies (*I*^2^ = 0).

### 3.2 Live birth rate

Nine studies reported live birth rate, and 9 studies were included in this meta-analysis (Fig. 3B). The meta-analysis showed that GH addition could significantly increase the live birth rate (RR 1.73, 95% CI 1.25–2.40; *P *< 0.001) per transfer cycle. There was no heterogeneity between studies (*I*^2^ = 0).

### 3.3 Cancelled cycles rate

Seven of the 11 studies^[15,20–24]^ reported the cancelled cycles rate in the meta-analysis (Fig. 3C). Pooling their results showed that the cancelled cycles rate (RR 0.65, 95% CI 0.45–0.94; *P* = 0.02) was significantly lower in patients who received GH. There was no heterogeneity among studies (*I*^2^ = 0).

### 3.4 Implantation rate

The implantation rate was reported in 5 studies, which were included in this meta-analysis (Fig. 3D). The pooled analysis demonstrated no significant difference in the implantation rate (RR 1.05, 95% CI 0.56–1.99; *P* = 0.87). There was high heterogeneity between the studies (*I*^2^ = 73%).

### 3.5 Fertilization rate

**Figure 4:**
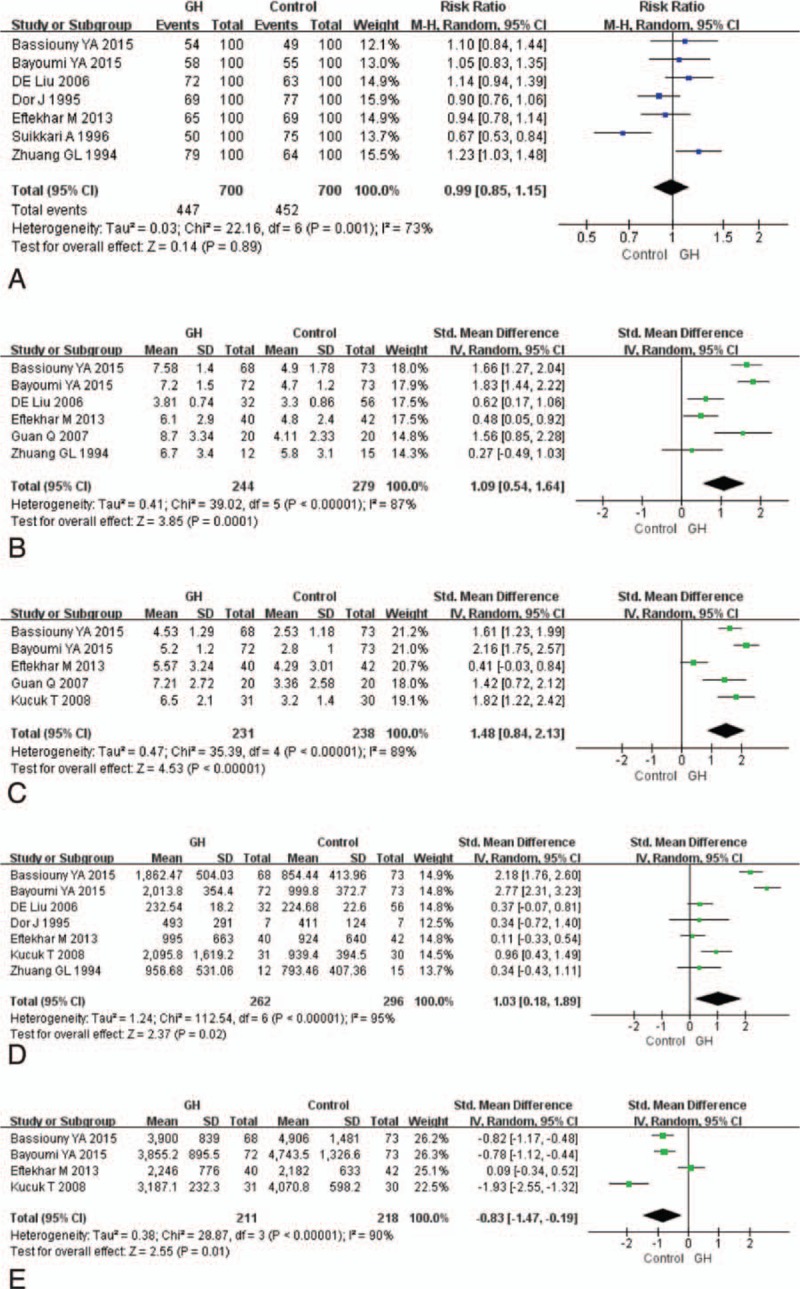
Forest plots for (A) fertilization rate, (B) collected oocytes number, (C) metaphase II oocyte number, (D)E_2_ on HCG day, and (E) dose of gonadotropin. CI = confidence interval, GH = growth hormone.

A total of 7 studies reported on the fertilization rate and were included in this meta-analysis (Fig. 4A). There was no significant difference between the GH group and the control group in the fertilization rate (RR 0.99, 95% CI 0.85–1.15; *P* = 0.89). High heterogeneity existed between the studies (*I*^2^ = 73%).

### 3.6 Collected oocytes number

Six studies reported collected oocytes number and were included in the meta-analysis (Fig. 4B). The pooled results indicated that the GH addition significantly increased collected oocytes number (SMD 1.09, 95% CI 0.54–1.64; *P *< 0.001). There was high heterogeneity between the studies (*I*^2^ = 87%).

### 3.7 MII oocyte number

Five studies reported MII oocytes number and were included in the meta-analysis (Fig. 4C). The pooled results indicated that the GH addition significantly increased MII oocytes number (SMD 1.48, 95% CI 0.84–2.13; *P *< 0.001). There was high heterogeneity between the studies (*I*^2^ = 89%).

### 3.8 E_2_ on HCG day

Seven studies reported E_2_ level on HCG day and were included in the meta-analysis (Fig. 4D). Pooling their results showed that E_2_ on HCG day (SMD 1.03, 95% CI 0.18–1.89; *P* = 0.02) was significantly higher in patients who received GH. High heterogeneity existed between the studies (*I*^2^ = 95%).

### 3.9 Dose of gonadotropin

Eight studies reported dose of gonadotropin but only 4 were included in the meta-analysis (Fig. 4E). Two studies used ampules as measure, which were different from other studies.^[22,25]^ Two studies used median was also excluded.^[18,23]^ The dose of gonadotropin (SMD –0.83, 95%CI –1.47, –0.19; *P* = 0.01) was significantly lower among patients who received GH than among those who was in the control group. There was high heterogeneity between the studies (*I*^2^ = 90%).

### 3.10 Subgroup analysis

**Figure 5:**
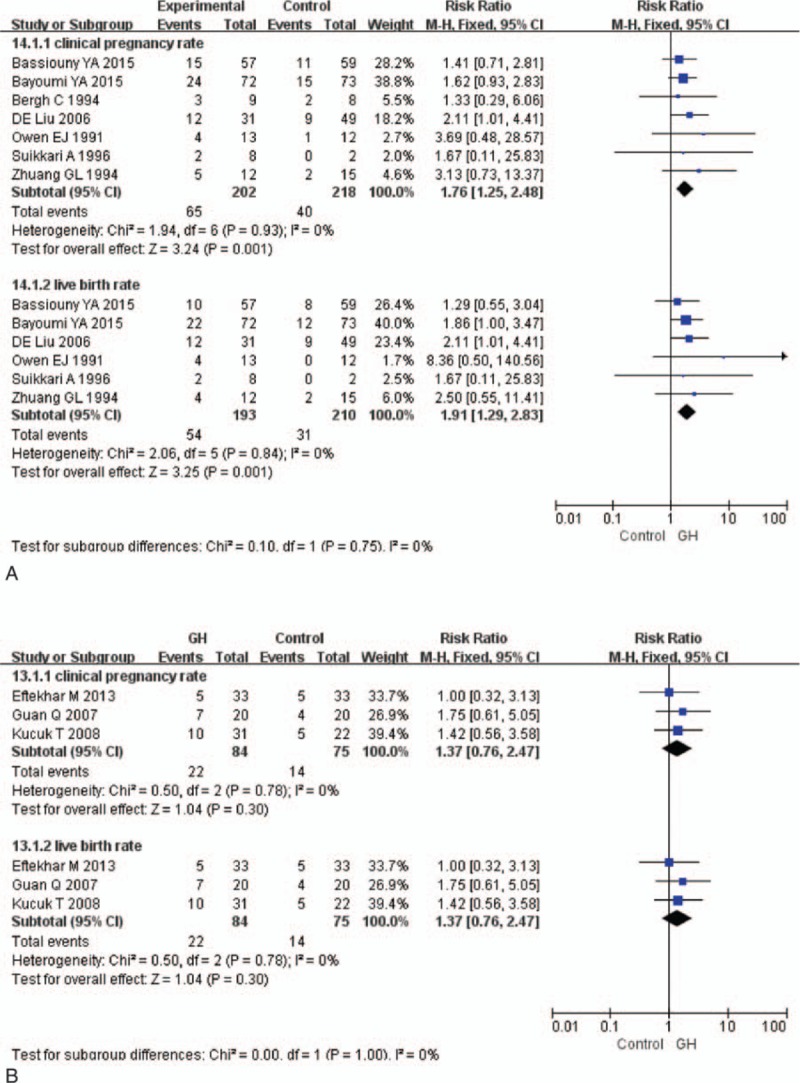
Forest plots for subgroup analysis (A) GH addition with Gn subgroup, and (B) GH addition in the middle luteal phase subgroup. CI = confidence interval, GH = growth hormone, Gn = Gonadotropin.

Seven articles^[15,16,18,19,20,22,23]^ were included in the GH addition with Gn group, clinical pregnancy rate (RR 1.76, 95% CI 1.25–2.48; *P* = 0.001) and live birth rate (RR 1.91, 95% CI 1.29–2.83; *P* = 0.001) was significantly increased in this group (Fig. 5A). There was no heterogeneity among studies (*I*^2^ = 0). Three articles^[17,21,24]^ were included in the GH addition in the middle luteal phase group, there were no significant differences for clinical pregnancy rate (RR 1.37, 95% CI 0.76–2.47; *P* = 0.30) (Fig. 5B) and live birth rate (RR 1.37, 95% CI 0.76–2.47; *P* = 0.30) (Fig. 5B) in the GH addition in the middle luteal phase group, there was no heterogeneity among studies (*I*^2^ = 0).

### 3.11 Adverse events

Only 1 study reported slight edema in 2 patients for a short period during treatment. Six studies reported no adverse effects during the process of studies, while the other 4 studies had no related information about the effect of GH addition.

## 4 Discussion

The present systematic review and meta-analysis of RCT demonstrated that co-treatment with GH in controlled ovary stimulation cycles significantly improved clinical pregnancy rate, live birth rate, collected oocytes number, MII oocytes number and E_2_ on HCG day in POR. Besides, cancelled cycles rate and dose of gonadotropin were significantly lower in patients who received the treatment of GH. There were no significant differences between the GH and control groups on the implantation rate and the fertilization rate. The subgroup analysis indicated that the GH addition with Gn group significantly increased the clinical pregnancy rate and the live birth rate, however, as for the clinical pregnancy rate and live birth rate at the GH addition in the middle luteal phase group, no significant differences were found.

GH plays an essential role in the function of ovarian, as it can stimulate the growth and function of granulose cells by increasing intraovarian production of insulin-like growth factor-1 (IGF-1).^[9]^ Research on animal and human have shown that GH is important for ovarian steroidogenesis and follicular development. Co-treatment with GH improves the Gn effects on granulose cells. Regarding the use of GH, a study showed that mouse oocyte maturation was significantly affected by treating with GH and IGF-1, respectively or collectively ^[26]^. A recent meta-analysis about different therapeutic protocols for ovarian stimulation of POR found that GH addition could improve clinical pregnancy rate and live birth rate, however the total numbers in the meta-analysis were small (251 patients) to draw any definitive conclusions.^[27]^ A review of 2009 about several interventions for patients with POR reported that GH addition appeared to improve the probability of pregnancy. In another meta-analysis,^[11]^ which included 6 RCT examined addition of GH to Gn in ovarian stimulation of POR and found that GH addition significantly increased the clinical pregnancy rate and live birth rate, as in the present study (11RCT). However, a meta-analysis by Yu et al reported that no significant difference was found for clinical pregnancy rate between the GH and control groups, which was not consistent with the present meta-analysis, the author speculate that it may be associated with the quality of the included articles (6 RCTs and 5 CCTs) or the difference of analysis methods.

A study which compared 4 stimulation protocols in POR with GH addition showed that number of retrieved and fertilized oocytes were highest in the long/GH protocol when compared in the rest of the protocols, while considering the clinical pregnancy rate, there was a difference for the long/GH protocol but the difference did not reach statistical significance.^[28]^ Some investigators had been confirmed low-dose GH supplementation increased clinical pregnancy rate in POR undergoing IVF.^[29]^ Another study showed the pregnancy rate was higher in the GH group than in the control group in patients with repeated IVF failures.^[30]^ In a sequential crossover study, GH supplementation improved implantation rate^[31]^ in poor-prognosis patients which is different from our result, we speculate that it may be connected with the different expression of rate. One study demonstrated GH addition significantly increased in the fertilization rate for those patients who had ICSI in GH deficiency patients.^[32]^ There was evidence that GH addition significantly lower cycle cancellations in POR with micro dose gonadotropin releasing hormone (GnRH) agonist protocol^[33]^ which was consistent with the present meta-analysis. However, retrospective matched case-control study reported there was no difference between the groups in clinical pregnancy rate and cycle cancellation rate in POR patients,^[34]^ which is different from our analysis. Result of Gregoraszczuk et al^[35]^ demonstrated that the influence of exogenous GH on steroid secretion by granulose cells and theca cells recovered from different follicles, GH addition stimulated both estradiol and progesterone secretion from large preovulatory follicles. However, Tapanainen et al^[36]^ suggested that serum E_2_ concentration was lower in the GH group than in the placebo group of HCG day for normally cycling women in vitro fertilization, which was not a finding of the present meta-analysis.

Potential limitation of the present study includes the inclusion of different dose of GH addition, and the different definition of POR. Furthermore, 2 articles are different from the other articles. One article had 4 groups, but only group I and group II were included, because group I is about GH use with standard protocol and group II is about standard treatment, Groups III and IV about GH preprocessing were eliminated. Another study included 3 groups, placebo, GH4 IU and GH 12 IU, as only 2 groups could be compared for the software, the 2 GH groups were merged and compared with placebo group in this meta-analysis. These 2 studies were analyzed separately and no significant difference in the overall result was recorded, so it was decided to add these 2 studies and analyze all 11 studies together.

Regarding to the heterogeneity of the included studies, there was high heterogeneity in the analysis except the pregnancy rate, live birth rate, and cancelled cycles rate. The sources of heterogeneity between the studies may be related to the different timings and doses of GH.

In summary, GH administration can improve the ovarian response in the patients with POR.^[37]^ The addition of GH significantly improved the clinical pregnancy rate, live birth rate, number of oocytes collected, MII oocyte number, and E_2_ on HCG day in POR. Besides, the cancelled cycles rate and dose of Gn were significantly lower in patients who received GH. No significant differences were found between the GH and control groups for the implantation rate and the fertilization rate. The subgroup analysis showed GH addition with the Gn group significantly increased the clinical pregnancy rate and the live birth rate. Furthermore, for the GH addition in the middle luteal phase group, no significant differences were found for the clinical pregnancy rate and the live birth rate. As the total number of patients analyzed in the GH addition with Gn group and the GH addition in the middle luteal phase group is small and further larger RCT with adequate simple sizes are needed to reach more definitive verdict.

## References

[1] Keay SD Liversedge NH Mathur RS Assisted conception following poor ovarian response to gonadotrophin stimulation. Brit J Obstet Gynaecol 1997;104:521–7. 9166190 10.1111/j.1471-0528.1997.tb11525.x

[2] Papathanasiou A Searle BJ King NMA Trends in ‘poor responder’ research: lessons learned from RCTs in assisted conception. Hum Reprod Update 2016;0:1–4. 10.1093/humupd/dmw001 26843539

[3] Sunkara SK Tuthill J Khairy M Pituitary suppression regimens in poor responders undergoing IVF treatment: a systematic review and meta-analysis. Reprod Biomed Online 2007;15:539–46. 18028745 10.1016/s1472-6483(10)60386-0

[4] Surrey ES Schoolcraft WB Evaluating strategies for improving ovarian response of the poor responder undergoing assisted reproductive techniques. Fertil Steril 2000;73:667–76. 10731523 10.1016/s0015-0282(99)00630-5

[5] Lashen H Ledger W Management of poor responders in IVF. Hum Reprod 1999;14:1919–21. 10402418 10.1093/humrep/14.7.1919

[6] Ferraretti AP La Marca A Fauser BCJM ESHRE consensus on the definition of ’poor response’ to ovarian stimulation for in vitro fertilization: the Bologna criteria. Hum Reprod 2011;26:1616–24. 21505041 10.1093/humrep/der092

[7] Ubaldi F Rienzi L Ferrero S Management of poor responders in IVF. Reprod Biomed Online 2005;10:235–46. 15823231 10.1016/s1472-6483(10)60946-7

[8] Magon N Agrawal S Malik S Growth hormone in the management of female infertility. Indian J Endocrinol Metab 2011;15 suppl 3:S246–7. 22029032 10.4103/2230-8210.84876 PMC3183519

[9] Bachelot A Monget P Imbert-Bollore P Growth hormone is required for ovarian follicular growth. Endocrinology 2002;143:4104–12. 12239122 10.1210/en.2002-220087

[10] Owen EJ West C Mason BA Co-treatment with growth hormone of sub-optimal responders in IVF-ET. Hum Reprod (Oxford, England) 1991;6:524–8. 10.1093/oxfordjournals.humrep.a137372 1918302

[11] Kolibianakis EM Venetis CA Diedrich K Addition of growth hormone to gonadotrophins in ovarian stimulation of poor responders treated by in-vitro fertilization: a systematic review and meta-analysis. Hum Reprod Update 2009;15:613–22. 19561136 10.1093/humupd/dmp026

[12] Kyrou D Kolibianakis EM Venetis CA How to improve the probability of pregnancy in poor responders undergoing in vitro fertilization: a systematic review and meta-analysis. Fertil Steril 2009;91:749–66. 18639875 10.1016/j.fertnstert.2007.12.077

[13] Yu X Ruan J He LP Efficacy of growth hormone supplementation with gonadotrophins in vitro fertilization for poor ovarian responders: an updated meta-analysis. Int J Clin Exp Med 2015;8:4954–67. 26131068 PMC4483949

[14] Harper K Proctor M Hughes E Growth hormone for in vitro fertilization. Cochrane Database Syst Rev 2003;Cd000099. 12917883 10.1002/14651858.CD000099

[15] Bayoumi YA Dakhly DR Bassiouny YA Addition of growth hormone to themicroflare stimulation protocol among women with poor ovarian response. Int J Gynecol Obstet 2015;131:305–8. 10.1016/j.ijgo.2015.05.034 26381201

[16] Zhuang GL Wong SX Zhou CQ The effect of co-administration of low dosage growth hormone and gonadotropin for ovarian hyperstimulation in vitro fertilization and embryo transfer. Zhonghua Fu Chan Ke Za Zhi 1994;29:471–4. 510. 7835118

[17] Guan Q Ma HG Wang YY Effects of co-administration of growth hormone(GH) and aspirin to women during in vitro fertilization and embryo transfer (IVF-ET) cycles. Zhonghua Nan Ke Xue = Nat J Androl 2007;13:798–800. 17929555

[18] Liu D Li Y Wu L Influence of adjuvant growth hormone therapy on in-vitro fertilization and embryo transplantation in poor responders of superovulation. J Clin Res 2006;23:1219–24.

[19] Owen EJ Shoham Z Mason BA Cotreatment with growth hormone, after pituitary suppression, for ovarian stimulation in in vitro fertilization: a randomized, double-blind, placebo-control trial. Fertil Steril 1991;56:1104–10. 1743329 10.1016/s0015-0282(16)54724-4

[20] Bassiouny YA Dakhly DM Bayoumi YA Does the addition of growth hormone to the in vitro fertilization/intracytoplasmic sperm injection antagonist protocol improve outcomes in poor responders? A randomized, controlled trial. Fertil Steril 2015;105:697–702. 26690008 10.1016/j.fertnstert.2015.11.026

[21] Kucuk T Kozinoglu H Kaba A Growth hormone co-treatment within a GnRH agonist long protocol in patients with poor ovarian response: a prospective, randomized, clinical trial. J Assist Reprod Genet 2008;25:123–7. 18392675 10.1007/s10815-008-9212-7 PMC2582075

[22] Suikkari A MacLachlan V Koistinen R Double-blind placebo controlled study: human biosynthetic growth hormone for assisted reproductive technology. Fertil Steril 1996;65:800–5. 8654642 10.1016/s0015-0282(16)58217-x

[23] Bergh C Hillensjo T Wikland M Adjuvant growth hormone treatment during in vitro fertilization: a randomized, placebo-controlled study. Fertil Steril 1994;62:113–20. 7516295

[24] Eftekhar M Aflatoonian A Mohammadian F Adjuvant growth hormone therapy in antagonist protocol in poor responders undergoing assisted reproductive technology. Arch Gynecol Obstetr 2013;287:1017–21. 10.1007/s00404-012-2655-1 23208461

[25] Dor J Seidman DS Amudai E Adjuvant growth hormone therapy in poor responders to in-vitro fertilization: a prospective randomized placebo-controlled double-blind study. Hum Reprod (Oxford, England) 1995;10:40–3. 10.1093/humrep/10.1.40 7745068

[26] Kiapekou E Loutradis D Drakakis P Effects of GH and IGF-I on the in vitro maturation of mouse oocytes. Hormones (Athens, Greece) 2005;4:155–60. 10.14310/horm.2002.11153 16613825

[27] Jeve YB Bhandari HM Effective treatment protocol for poor ovarian response: a systematic review and meta-analysis. J Hum Reprod Sci 2016;9:70–81. 27382230 10.4103/0974-1208.183515 PMC4915289

[28] Dakhly DM Bayoumi YA Gad Allah SH Which is the best IVF/ICSI protocol to be used in poor responders receiving growth hormone as an adjuvant treatment? A prospective randomized trial. Gynecol Endocrinol 2015;32:1–4. 26416521 10.3109/09513590.2015.1092136

[29] Lattes K Brassesco M Gomez M Low-dose growth hormone supplementation increases clinical pregnancy rate in poor responders undergoing in vitro fertilisation. Gynecol Endocrinol 2015;31:565–8. 26193891 10.3109/09513590.2015.1025378

[30] Hazout A Junca A Menezo Y Effect of growth hormone on oocyte competence in patients with multiple IVF failures. Reprod Biomed Online 2009;18:664–70. 19549445 10.1016/s1472-6483(10)60011-9

[31] Yovich JL Stanger JD Growth hormone supplementation improves implantation and pregnancy productivity rates for poor-prognosis patients undertaking IVF. Reprod Biomed Online 2010;21:37–49. 20457541 10.1016/j.rbmo.2010.03.013

[32] Rajesh H Yong YY Zhu M Growth hormone deficiency and supplementation at in-vitro fertilisation. Singapore Med J 2007;48:514–8. 17538748

[33] Schoolcraft W Schlenker T Gee M Improved controlled ovarian hyperstimulation in poor responder in vitro fertilization patients with a microdose follicle-stimulating hormone flare, growth hormone protocol. Fertil Steril 1997;67:93–7. 8986690 10.1016/s0015-0282(97)81862-6

[34] Dunne C Seethram K Roberts J Growth hormone supplementation in the luteal phase before microdose GnRH agonist flare protocol for in vitro dertilization. J Obstet Gynaecol Canada 2015;37:810–5. 10.1016/S1701-2163(15)30152-3 26605451

[35] Gregoraszczuk EL Bylica A Gertler A Response of porcine theca and granulosa cells to GH during short-term in vitro culture. Anim Reprod Sci 2000;58:113–25. 10700649 10.1016/s0378-4320(99)00083-4

[36] Tapanainen J Martikainen H Voutilainen R Effect of growth hormone administration on human ovarian function and steroidogenic gene expression in granulosa-luteal cells. Fertil Steril 1992;58:726–32. 1426317 10.1016/s0015-0282(16)55319-9

[37] de Ziegler D Streuli I Meldrum DR The value of growth hormone supplements in ART for poor ovarian responders. Fertil Steril 2011;96:1069–76. 22036051 10.1016/j.fertnstert.2011.09.049

